# Simultaneous Calibration of the Hand-Eye, Flange-Tool and Robot-Robot Relationship in Dual-Robot Collaboration Systems

**DOI:** 10.3390/s22051861

**Published:** 2022-02-26

**Authors:** Yanding Qin, Pengxiu Geng, Bowen Lv, Yiyang Meng, Zhichao Song, Jianda Han

**Affiliations:** 1 Tianjin Key Laboratory of Intelligent Robotics, College of Artificial Intelligence, Nankai University, Tianjin 300350, China; 1120210170@mail.nankai.edu.cn (P.G.); 1120200176@mail.nankai.edu.cn (B.L.); 2120200374@mail.nankai.edu.cn (Y.M.); 2120180405@mail.nankai.edu.cn (Z.S.); hanjianda@nankai.edu.cn (J.H.); 2 Institute of Intelligence Technology and Robotic Systems, Shenzhen Research Institute of Nankai University, Shenzhen 518083, China

**Keywords:** robot calibration, **AXB**=**YCZ**, dual quaternion, Levenberg–Marquardt algorithm

## Abstract

A multi-robot collaboration system can complete more complex tasks than a single robot system. Ensuring the calibration accuracy between robots in the system is a prerequisite for the effective inter-robot cooperation. This paper presents a dual-robot system for orthopedic surgeries, where the relationships between hand-eye, flange-tool, and robot-robot need to be calibrated. This calibration problem can be summarized to the solution of the matrix equation of **AXB**=**YCZ**. A combined solution is proposed to solve the unknown parameters in the equation of **AXB**=**YCZ**, which consists of the dual quaternion closed-form method and the iterative method based on Levenberg–Marquardt (LM) algorithm. The closed-form method is used to quickly obtain the initial value for the iterative method so as to increase the convergence speed and calibration accuracy of the iterative method. Simulation and experimental analyses are carried out to verify the accuracy and effectiveness of the proposed method.

## 1. Introduction

A multi-robot collaboration system is frequently used to complete tasks that are very difficult or even impossible for a single robot system. It has the advantages of high precision, more complex tasks, high reliability, etc. Therefore, it is widely used in medical [1], aerospace [2] and other fields.

Robot and navigation technologies have been successfully integrated in the robotic orthopedic surgery system, where a robot is utilized to assist or even replace the surgeon during the intraoperative bone cutting with the position feedback from an optical tracking system (OTS). Generally, the pose of the OTS can be manually adjusted by the surgeon before the surgery and stays stationary throughout the surgery. However, the measurement accuracy of the OTS is related to the relative position and orientation between the OTS and the target [3,4]. In orthopedic surgeries, OTS is used to simultaneous locate multiple targets, such as the robot, the surgical tool, and the patient’s bone. It is difficult for the surgeon to manually move the OTS to the optimal pose for the best measurement accuracy for all the targets. Furthermore, the operation room is crowded, especially around the operation table. As a result, the surgeon, the staff or the medical instruments might interfere with the measurement volume of the OTS. If the OTS is fixed, occlusions are likely to occurs, leading to the cut off of the feedback from the OTS. This is fatal in robotic surgery systems.

In this paper, a dual-robot system is developed for orthopedic surgeries, where an extra robot, i.e., the navigation robot, is used to carry the OTS such that the pose of the OTS can be actively adjusted during the surgical operation. The benefits for this active navigation include: (1) for multiple targets, the optimal pose of the OTS can be calculated according to the poses of each target, and then the navigation robot can precisely adjust the OTS to the optimal pose so as to guarantee the precision of the overall system, and (2) the navigation robot can move the OTS before the occlusion occurs so as to keep all the targets in sight throughout the surgery.

**Figure 1:**
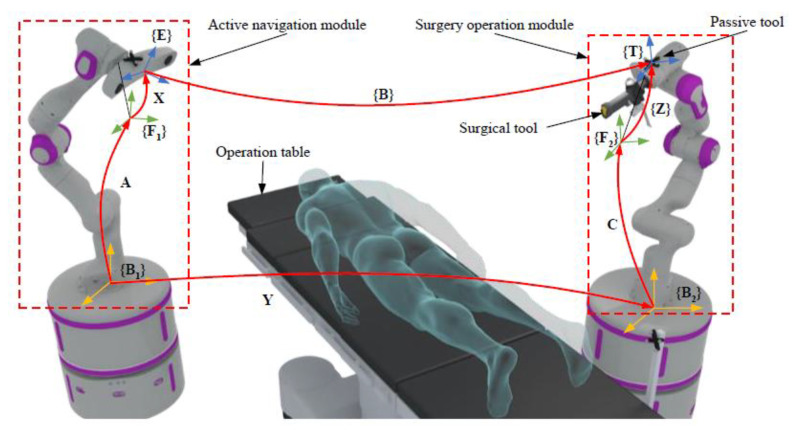
The schematic diagram of the dual-robot system developed for orthopedic surgeries, where the patient is facing down for spine surgery.

As schematically shown in Figure 1, the dual-robot system consists of an active navigation module and a surgery operation module located on both sides of the operation table. The active navigation module is composed of an OTS fixed to the flange of the navigation robot. In the surgery operation module, the surgical tool and a passive tool are fixed to the flange of the operation robot.

The prerequisite for the collaborative operation of the dual-robot system is that the two robots, the OTS and the surgical tool are unified under the same world coordinate system. As shown in Figure 1, the following homogeneous transformation matrices (HTMs) are defined: **A** is the HTM from the base of the navigation robot {B_1_} to its flange {F_1_}, **B** is the HTM from the OTS {E} to the passive tool {T}, **C** is the HTM from the base {B_2_} to its flange {F_2_} of the operation robot, **X** is the HTM from {F_1_} to {E}, **Y** is the HTM from {B_1_} to {B_2_}, and **Z** is the HTM from {F_2_} to {T}. Therefore, the calibration problem of the dual-robot system can be summarized to the solution of the following equation:

(1) AXB=YCZ,

where **A**, **B**, and **C** are known, and **X**, **Y**, and **Z** are the parameters to be calibrated.

For different robot configurations, the calibration algorithm can be briefly divided into three categories:

(1) **AX**=**XB**. It aims to solve the robot hand-eye relationship matrix **X** under the condition that both **A** and **B** are known. Shiu first proposed a method to solve the matrix equation in the hand-eye calibration problem [5]. Tsai proposed an analytical method to solve the problem of hand-eye calibration and gave the principle of selecting the data set [6]. Fassi analyzed the algorithm for solving the hand-eye calibration matrix formula from a geometric point of view [7]. At the same time, they studied the excessive restriction and singularity in the algorithm. Other algorithms include dual quaternion method [8], Kronecker product method [9], screw theory method [10], optimization methods [11], etc.

(2) **AX**=**YB**. This focuses on how to solve the problem of **X** and **Y** simultaneously under the condition of known **A** and **B**. Zhuang first presented a linear solution that allows a simultaneous computation of the transformations from robot world to robot base and from robot tool to robot flange coordinate frames [12]. Tan used three calibration methods based on nonlinear optimization and evolutionary computation to calibrate the underactuated robotic hand with soft fingers [13]. Condurache designed the simultaneous closed-form solutions for the sensor calibration problem by using an isomorphism between the special Euclidean group SE(3) and the orthogonal dual tensors group SO(3), which are based on the properties of the orthogonal dual tensors [14]. Wu presented a new error formulation using Cayley transform, which allowed for a unified approach for **AX**=**XB** and **AX**=**YB** simultaneously [15].

(3) **AXB**=**YCZ**. This is to solve the problem of multi-robot system calibration, which is also the research topic of this paper. Jin proposed a non-contact hand-eye calibration method for dual robot vision system, which was used by both machines to improve the accuracy of coordinate reading [16]. Qiao proposed a novel method for multi-robot system calibration utilizing a calibration model based on the Product of Exponentials formula without nominal kinematic parameters. With this method, the robot nominal kinematic model reduces the requirements of kinematic parameters, which also simplifies the modeling process [17]. These above methods are in common that **X**, **Y**, and **Z** are obtained step-by-step, which might cause the accumulation of the errors in each step, resulting in unreliable calibration results.

In order to reduce the error of the above step-by-step calibration methods, many researchers have studied the method of simultaneous calibration. Wang first proposed a simultaneous hand-eye, flange-tool, and robot-robot calibration method to obtain the relationships of multiple robots, where a linear approximation iterative algorithm was used [18]. On the basis of Wang’s method, Wu added the closed-form method based on quaternion as the initial value of the iterative method to improve its calibration accuracy [19]. Yan proposed a Degradation-Kronecker (DK) method as an optimal closed-form solution for the registration of a hybrid robot and used a purely nonlinear method to complement the DK method [20]. Ma proposed two probabilistic approaches that can solve the **AXB**=**YCZ** problem for the case where the correspondence information between the datasets was not required [21]. However, measurement errors were not explicitly modeled, thus the results were sensitive to the measurement noise. Therefore, Ma proposed a method to increase the robustness against noise, including a hybrid method that combines traditional **AXB**=**YCZ** solvers with probabilistic methodology and an iterative method for the refinement to increase the robustness in the case of noisy experimental data [22]. Fu proposed a closed-form method based on dual quaternion to solve the calibration problem, which effectively improved the calibration accuracy [23]. Wang proposed a novel approach that is composed of a closed-form method based on the Kronecker product and an iterative method that converts the calculation of a nonlinear problem to an optimization problem of a strictly convex function [24].

In this paper, in order to obtain the THMs of hand-eye, flange-tool, and robot-robot, a novel approach for simultaneously calibrating the unknown parameters **X**, **Y**, **Z** of **AXB**=**YCZ** is proposed. The method is composed of a dual quaternion based closed-form method and a Levenberg Marquardt (LM) algorithm based iterative method. Since the result of the iterative method is greatly affected by the initial value, the purpose of the closed-form method is to generate a credible initial value, which makes the iterative method converge faster and improve its calibration accuracy.

The following of this paper is organized as follows. Section 2 introduces the basic knowledge of the dual quaternion, modeling and formulating the calibration problem, and then introduces the calibration method proposed in this paper. The simulation and experimental verifications are illustrated in Section 3. Ultimately, the conclusions are provided in Section 4.

## 2. Materials and Methods

### 2.1. Dual Quaternion

Dual quaternion is proposed on the basis of quaternion and dual geometric algebra theory, which can solve general rigid body motion (rotation and translation) problems. The dual quaternion consists of two parts:

(2) $$\hat{q}=q+\varepsilon q^{\prime }={\left(q_{0},\;q_{1},\;q_{2},\;q_{3}\right)}^{T}+\varepsilon {\left(q_{00},\;q_{01},\;q_{02},\;q_{03}\right)}^{T},$$

where $\hat{q}$ is a dual quaternion; *q* and $q^{\prime }$ are two quaternions, which are called the real part and the dual part of $\hat{q}$, respectively; and *ε* is the dual operator, which satisfies the property of *ε*^2^ = 0 and *ε* ≠ 0. The basic operations for two dual quaternions are given in Table 1.

If $\left\|\hat{q}\right\|$ = 1, the dual quaternion $\hat{q}$ is a unit dual quaternion satisfying the following constraints:

(3) $$\{\begin{array}{c} q^{T}q=1,\\ q^{T}q^{\prime }=0. \end{array}$$

The multiplication of two quaternions is defined in the following equation:

(4) $$\begin{array}{c} p\cdot q=(p_{0}q_{0}-{\vec{p}}^{T}\vec{q})+(p_{0}\vec{q}+q_{0}\vec{p}+\vec{p}\times \vec{q})\\ \;=Q_{L}(p)\cdot q=Q_{R}(q)\cdot p. \end{array}$$

where $Q_{L}(p)=\left[\begin{array}{cccc} p_{0} & -p_{1} & -p_{2} & -p_{3}\\ p_{1} & p_{0} & -p_{3} & p_{2}\\ p_{2} & p_{3} & p_{0} & -p_{1}\\ p_{3} & -p_{2} & p_{1} & p_{0} \end{array}\right],$and $Q_{R}(q)=\left[\begin{array}{cccc} q_{0} & -q_{1} & -q_{2} & -q_{3}\\ q_{1} & q_{0} & q_{3} & -q_{2}\\ q_{2} & -q_{3} & q_{0} & q_{1}\\ q_{3} & q_{2} & -q_{1} & q_{0} \end{array}\right]$.

### 2.2. The Closed-Form Calibration Method

In 3D space, the motion of a rigid body is the rotation and translation of the coordinates around the axes. The 4 × 4 HTM **T** is generally used to describe the kinematics model of the rigid body:

(5) $$T=\left[\begin{array}{cccc} r_{11} & r_{12} & r_{13} & t_{x}\\ r_{21} & r_{22} & r_{23} & t_{y}\\ r_{31} & r_{32} & r_{33} & t_{z}\\ 0 & 0 & 0 & 1 \end{array}\right]=\left[\begin{array}{cc} R & t\\ 0 & 1 \end{array}\right],$$

where **R** is the rotation matrix and **t** is the translation vector.

Similarly, the unit dual quaternion can also represent the rotation and translation of a rigid body in the 3D space. For a given HTM, the corresponding unit dual quaternion can be expressed as $\hat{q}$ = *q* + *ε* $q^{\prime }$ , and the real part and dual part can be calculated using:

(6) $$\begin{array}{c} q_{0}=\sqrt{tr(R)+1}/2,\\ q_{1}=(r_{32}-r_{23})/4q_{0},\\ q_{2}=(r_{13}-r_{31})/4q_{0},\\ q_{3}=(r_{21}-r_{12})/4q_{0},\\ q={\left(q_{0},\;q_{1},\;q_{2},\;q_{3}\right)}^{T},\\ q^{\prime }={\left(\frac{1}{2}\left[\begin{array}{ccc} -q_{1} & -q_{2} & -q_{3}\\ q_{0} & q_{3} & -q_{2}\\ -q_{3} & q_{0} & q_{1}\\ q_{2} & -q_{1} & q_{0} \end{array}\right]t\right)}^{T}. \end{array}$$

Accordingly, for a given unit dual quaternion, the corresponding HTM can be expressed as follows:

(7) $$R=\left[\begin{array}{ccc} q_{0}^{2}+q_{1}^{2}-q_{2}^{2}-q_{3}^{2} & 2(q_{1}q_{2}-q_{0}q_{3}) & 2(q_{1}q_{3}+q_{0}q_{2})\\ 2(q_{1}q_{2}+q_{0}q_{3}) & q_{0}^{2}-q_{1}^{2}+q_{2}^{2}-q_{3}^{2} & 2(q_{2}q_{3}-q_{0}q_{1})\\ 2(q_{1}q_{3}-q_{0}q_{2}) & 2(q_{2}q_{3}+q_{0}q_{1}) & q_{0}^{2}-q_{1}^{2}-q_{2}^{2}+q_{3}^{2} \end{array}\right],$$

(8) $$t=2\left[\begin{array}{cccc} -q_{1} & q_{0} & -q_{3} & q_{2}\\ -q_{2} & q_{3} & q_{0} & -q_{1}\\ -q_{3} & -q_{2} & q_{1} & q_{0} \end{array}\right]\left[\begin{array}{c} q_{00}\\ q_{01}\\ q_{02}\\ q_{03} \end{array}\right].$$

According to the work of Daniilidis [8], HTMs and dual quaternions are equivalent, and these two forms can be converted into each other. According to the conversion relationship between the HTM and the dual quaternion shown in (5) and (6), the equation in (1) can be rewritten using the dual quaternion as:

(9) $$\begin{array}{c} AXB=YCZ,\\ ⇓\;⇓\\ {\hat{q}}_{A}{\hat{q}}_{X}{\hat{q}}_{B}={\hat{q}}_{Y}{\hat{q}}_{C}{\hat{q}}_{Z}. \end{array}$$

where subscripts indicate the corresponding equivalent dual quaternions for the HTMs. Therefore, the solving problem of (1) is transformed into the solving problem of (9). The closed-form method for the kinematics calibration proceeds as follows.

#### 2.2.1. Determination of **Z_0_**

In order to determine the coordinate relationship between the passive tool frame {T} and the flange of the operation robot {F_2_} (as shown in Figure 1), it is assumed that the navigation robot remains stationary. Hence, it can be regarded as a hand-eye calibration problem. The operation robot is given *m* sets (*m* ≥ 3) of motions, and **Z_0_** can be calculated using the hand-eye calibration method proposed by Tsai at al. [6].

#### 2.2.2. Determination of **X_0_** and **Y_0_**

With the calibrated **Z_0_**, (9) can be rewritten to be:

(10) $${\hat{q}}_{A}{\hat{q}}_{X}={\hat{q}}_{Y}{\hat{q}}_{M},$$

where $\hat{q}$ *_M_* = $\hat{q}$*_C_* $\hat{q}$*_Z_* $\hat{q}$*_B_*^−1^. According to the multiplication rule of dual quaternion, the following equations can be obtained:

(11) $$\begin{array}{c} {\hat{q}}_{A}{\hat{q}}_{X}-{\hat{q}}_{Y}{\hat{q}}_{M}=0,\\ q_{A}q_{X}-q_{Y}q_{M}=0,\\ {q^{\prime }}_{A}q_{X}+q_{A}{q^{\prime }}_{X}-({q^{\prime }}_{Y}q_{M}+q_{Y}{q^{\prime }}_{M})=0,\\ G\cdot \Gamma =0, \end{array}$$

where $\Gamma ={\left[q_{X},{q^{\prime }}_{X},q_{Y},{q^{\prime }}_{Y}\right]}^{T}$ and $G=\left[\begin{array}{cccc} Q_{L}(q_{A}) & 0 & -Q_{R}(q_{M}) & 0\\ Q_{L}({q^{\prime }}_{A}) & Q_{L}(q_{A}) & -Q_{R}({q^{\prime }}_{M}) & -Q_{R}(q_{M}) \end{array}\right]$.

Let the navigation robot and the operation robot move at the same time and ensure that the OTS can track the passive tool at every point when two robots stop. Given *n* sets (*n* ≥ 3) of acquired data, the following 8*n* × 16 matrix is constructed:

(12) $$\Lambda ={\left[G_{1},G_{2},G_{3},\ldots ,G_{n}\right]}^{T}.$$

When the data are noise-free, rank(**Λ**) ≤ 14. The singular value decomposition (SVD) of **Λ** can be obtained:

(13) $$\Lambda =U\Sigma V^{T},$$

where **U** denotes an 8*n* × 8*n* orthogonal matrix, **Σ** is an 8*n* × 16 singular-value matrix and its diagonal elements are the eigenvalues of **Λ** in descending order, and the other elements are zero. Additionally, **V** represents a 16 × 16 orthogonal matrix, and each of its columns is composed of the eigenvectors of **Λ**. The last two column vectors, *v*_15_ and *v*_16_ in **V**, correspond to the singular values that are equal to zero, which span the null space of **Λ**.

Subsequently, **Γ** is a null vector of **Λ** and can be expressed as follows:

(14) $$\Gamma ={\left[q_{X},\;{q^{\prime }}_{X},\;q_{Y},\;{q^{\prime }}_{Y}\right]}^{T}=\eta_{1}v_{15}+\eta_{2}v_{16},$$

where *v*_15_ = [*a*_11_, *a*_12_, *a*_13_, *a*_14_]*^T^*, *v*_16_ = [ *a*_21_, *a*_22_, *a*_23_, *a*_24_]*^T^.* Note that $\hat{q}$ *_X_* =*q_X_* + ${\hat{q}}^{\prime }$*_X_*, $\hat{q}$*_Y_* = *q_Y_* + ${\hat{q}}^{\prime }$*_Y_*. According to the constraint of unit dual quaternion, we can obtain:

(15) $$\{\begin{array}{c} \eta_{1}{}^{2}a_{11}{}^{T}a_{11}+2\eta_{1}\eta_{2}a_{11}{}^{T}a_{21}+\eta_{2}{}^{2}a_{21}{}^{T}a_{21}=1\\ \eta_{1}{}^{2}a_{11}{}^{T}a_{12}+\eta_{1}\eta_{2}(a_{11}{}^{T}a_{22}+a_{21}{}^{T}a_{12})+\eta_{2}{}^{2}a_{21}{}^{T}a_{22}=0. \end{array}$$

(16) $$\{\begin{array}{c} \eta_{1}{}^{2}a_{13}{}^{T}a_{13}+2\eta_{1}\eta_{2}a_{13}{}^{T}a_{23}+\eta_{2}{}^{2}a_{23}{}^{T}a_{23}=1\\ \eta_{1}{}^{2}a_{13}{}^{T}a_{14}+\eta_{1}\eta_{2}(a_{13}{}^{T}a_{24}+a_{23}{}^{T}a_{14})+\eta_{2}{}^{2}a_{23}{}^{T}a_{24}=0. \end{array}$$

By solving the above equations, **X_0_** and **Y_0_** can be obtained.

Since the closed-form method does not require iterative calculations, its computation speed is very fast. However, the closed-form method has two obvious disadvantages:

(1) **Z_0_** and **X_0_**, **Y_0_** are calculated in a step-by-step manner. Therefore, the error of **Z_0_** will be accumulated in the calculation of **X_0_** and **Y_0_**.

(2) Since the closed-form method simply treats the nonlinearly constrained problem as a linear unconstrained problem, the calibration accuracy will be affected.

Therefore, the closed-form method is more suitable for the rapid calculation of the initial estimation of the iterative method to improve its accuracy and calculation efficiency.

### 2.3. The Iterative Calibration Method

In the iterative method, the rotational part and translational part are separately solved for the three unknown parameters in (1). By expanding the HTM, the following two equations can be derived:

(17) $$R_{A}R_{X}R_{B}=R_{Y}R_{C}R_{Z}.$$

(18) $$R_{A}R_{X}t_{B}+R_{A}t_{X}+t_{A}=R_{Y}R_{C}t_{Z}+R_{Y}t_{C}+t_{Y}.$$

#### 2.3.1. Solution of the Rotational Parts

LM algorithm [25,26,27] combines the characteristics of local convergence of Gauss–Newton method and the global search of the reduced gradient method. In this paper, LM algorithm is used to iteratively solve the rotational part in (17).

As can be seen from Figure 1, the geometric meaning of (1) is the homogeneous transformation matrix from the passive tool {**T**} to the base of the navigation robot {**B_1_**}. For a rotation matrix, it has the following properties:

(19) $$\begin{array}{l} R^{-1}=R^{T},\\ R^{T}\cdot R=I_{3}. \end{array}$$

For *n* sets of dual-robot poses, the error items of (17) can be defined according to the properties of the rotation matrix:

(20) $$p_{0}=\left[R_{X_{0}},R_{Y_{0}},R_{Z_{0}}\right].$$

(21) $$f_{i}(p)=\min\left[diag\left(R_{Ai}R_{X}R_{Bi}*R_{Y}R_{Ci}R_{Z}\right)-I_{3}\right],$$

where **I_3_** is a third-order unit matrix, and *i* = 1, 2, …, *n*. Hence, solving for the rotational part is equivalent to minimizing for the following objective function:

(22) $$F(p)=\frac{1}{2}\sum_{i=1}^{n}{\left(f_{i}(p)\right)}^{2}.$$

Using the LM algorithm to perform iterative operations, the rotational part can be obtained. The pseudocode of the LM Algorithm 1 is as follows:

**Algorithm** **1:** LM algorithm.Input: Maximum number of iterations *k_max_* = 100, *ε*_2_ = 10^−15^, *τ* = 10^−5^, The initial rotational part of, **X_0_**, **Y_0_**, **Z_0_**: **R**_*X*0_, **R**_*Y*0_, **R**_*Z*0_.Begin

$\;k≔0,\;v≔2,\;p≔p_{0}$



$\;A≔J{\left(p\right)}^{T}J\left(p\right),\;g≔J{\left(p\right)}^{T}f\left(p\right)$



$\;found≔\left({\left\|g\right\|}_{\infty }<\varepsilon_{1}\right),\mu ≔\tau *\max\left\{a_{ii}\right\}$



$\;while\;(notfound)\;and\;(k<k_{max})$



$\;k≔k+1,\;Solve\left(A+\mu I\right)h_{lm}=-g$



$\;if\left\|h_{lm}\right\|\leq \varepsilon_{2}\left(\left\|p\right\|+\epsilon_{2}\right)$



       found≔true 



     else



$\;p_{new}≔p+h_{lm}$



$\;\;\varrho ≔\left(F\left(p\right)-F\left(p_{new}\right)\right)/\left(L\left(0\right)-L\left(h_{lm}\right)\right)$



       ifϱ>0



$\;\;p≔p_{new}$



$\;A≔J{\left(p\right)}^{T}J\left(p\right),\;g≔J{\left(p\right)}^{T}f\left(p\right)$



$\;found:\left({\left\|g\right\|}_{\infty }<\varepsilon_{1}\right)$



$\;\mu ≔\mu *max\left\{\frac{1}{3},1-{\left(2\varrho -1\right)}^{3}\right\},v≔2\;$



       else



         μ≔μ∗v,v≔2∗v 



  end



#### 2.3.2. Solution of the Translational Parts

Equation (18) can be rewritten to be:

(23) Jt=P,

where **J** = [**R**_*A*_, −**I**, −**R**_*Y*_**R**_*C*_], **t** = [**t**_*X*_; **t**_*Y*_; **t**_*Z*_], **P**= −**t**_*A*_−**R**_*A*_**R**_*X*_**R**_*B*_ + **R**_*Y*_**t**_*C*_. Then, we have:

(24) $$J_{S}t=P_{S},$$

where $J_{S}=[J_{1};\;J_{2};\ldots ;\;J_{n}],\;P_{S}=[P_{1};\;P_{2};\ldots ;\;P_{n}].$ Therefore, **t** can be solved to be:

(25) $$t={\left(J_{S}{}^{T}J_{S}\right)}^{-1}J_{S}{}^{T}P_{S}.$$

## 3. Results

### 3.1. Simulation Analyses

Simulations were performed to evaluate the performance of the proposed method. A simulation environment was developed in MATLAB, where two 6-DOF robots (model Puma560) were used to construct the dual-robot cooperation system. The kinematic parameters of robots were derived from the robotics toolbox in MATLAB. The true values of **X**, **Y**, and **Z** in the simulation were assigned as follows:

(26) $$X_{true}=\left[\begin{array}{cccc} -0.0998 & -0.9950 & 0 & 0.05\\ 0.9950 & -0.0998 & 0 & -0.1\\ 0 & 0 & 1 & 0.05\\ 0 & 0 & 0 & 1 \end{array}\right],$$

(27) $$Y_{true}=\left[\begin{array}{cccc} -0.9801 & -0.1987 & 0 & 2.000\\ -0.1987 & -0.9801 & 0 & -0.3\\ 0 & 0 & 1 & 0.3\\ 0 & 0 & 0 & 1 \end{array}\right],$$

(28) $$Z_{true}=\left[\begin{array}{cccc} 0.4110 & -0.9116 & 0 & -0.05\\ -0.9116 & 0.4110 & 0 & 0.1\\ 0 & 0 & 1 & -0.05\\ 0 & 0 & 0 & 1 \end{array}\right].$$

For the proposed method in this paper, the following data sets were generated to complete the calibration process:

(1) Obtain *k* (*k* = 30) sets of data to calculate **Z_0_** using the traditional hand-eye calibration method [6]. Specifically, the operation robot moves within the measurement volume of the OTS and the navigation robot is stationary, i.e., **A** is constant. In this case, 30 different configurations of the operation robot were randomly generated, i.e., a group of 30 different **C** were obtained. Subsequently, the data of **B** were calculated according to the traditional hand-eye calibration method.

(2) Obtain another *m* (*m* = 120) sets of data used for the remaining calibration. The data of **A** and **C** were randomly generated within the workspace of the robot under the premise that the OTS can always track the passive tool. The data of B were calculated:

(29) $$B_{true}={\left(AX_{true}\right)}^{-1}Y_{true}CZ_{true}=\left[\begin{array}{cc} R_{B\_true} & t_{B\_true}\\ 0 & 1 \end{array}\right],$$

where **R***_B_true_* = Rot (*z*, *α_true_*) Rot (*y*, *β_true_*) Rot (*x*, *γ_true_*), and **t***_B_true_* = Trans (*t_x_true_*, *t_y_true_*, *t_z_true_*,).

In real applications, measurement noise is inevitable in the position and orientation feedback system, especially for the widely used OTS. To verify the robustness of the proposed method against the measurement noise, the noise level shown in Table 2 was added to all the generated data. In addition, in order to evaluate whether the size of the training data has an impact on the calibration results, *n* sets of training data (*n* = 10, 20, …, 120) were used for calibration in turns.

In order to quantitatively describe the calibration accuracy, define:

(30) $$B_{cal}={\left(A_{noise}X_{cal}\right)}^{-1}Y_{cal}C_{noise}Z_{cal}=\left[\begin{array}{cc} R_{B\_cal} & t_{B\_cal}\\ 0 & 1 \end{array}\right],$$

where **R***_B_cal_* = Rot (*z*, *α_cal_*) Rot (*y*, *β_cal_*) Rot (*x*, *γ_cal_*) and **t***_B_cal_* = Trans (*t_x_cal_*, *t_y_cal_*, *t_z_cal_*,). The following rotation error and translation error were adopted:

(31) $$\left\{ \begin{array}{l} R_{i_{ERROR}}=R_{B}{}_{i_{cal}}-R_{B}{}_{i_{true}},i=\alpha ,\beta ,\gamma .\\ t_{i_{ERROR}}=t_{B}{}_{i_{cal}}-t_{B}{}_{i_{true}},i=x,y,z. \end{array}\right.$$

**Figure 2:**
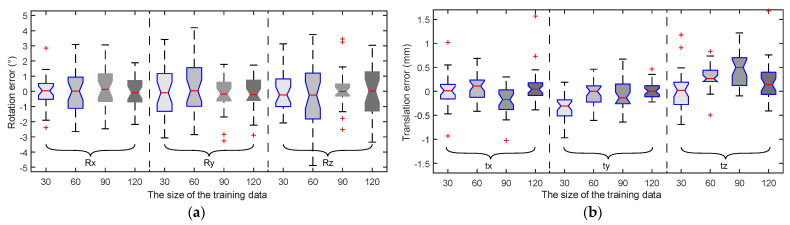
The statistics of the calibration error against different sizes of training data: (**a**) rotation error and (**b**) translation error. The red “+” signs are the outliers in the dataset.

In general, it has become a common practice to increase the size of the training data so as to increase the calibration accuracy. The proposed method was utilized to repeat the calibration using different amount of training data. A boxplot was utilized to evaluate the relationship between the translation and rotation errors and the size of the training data. As shown in Figure 2, it can be observed that the calibration accuracy is not very sensitive to the size of the training data. This implies that the proposed method is effective even with a small data set, and thus facilitating the real implementation.

### 3.2. Experimental Analyses

**Figure 3:**
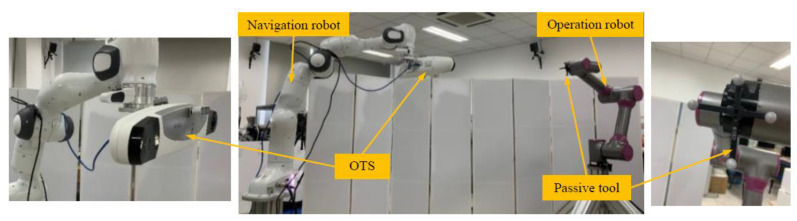
The experimental setup of the dual-robot system.

In order to experimentally verify the effectiveness of the proposed method, a calibration was conducted on the prototype of a dual-robot collaboration system developed for robotic orthopedic surgeries. As shown in Figure 3, the prototype consisted of a 7-DOF navigation robot (model Panda from Franka Emika), an in-house built 6-DOF operation robot, and an OTS (model Polaris Vega from NDI). The OTS was installed at the flange of the navigation robot, and a passive tool was installed at the flange of the operation robot.

This dual-robot system was calibrated using the proposed method. Firstly, 30 measurements were acquired for calculating **Z_0_**. The operation robot moved within the measurement volume of the OTS and the navigation robot was stationary. According to the hand-eye calibration method proposed by Tsai [6], the HTM of **Z_0_** was identified to be:

(32) $$Z_{0}=\left[\begin{array}{cccc} -0.7794 & -0.3853 & 0.4940 & -0.0606\\ 0.6265 & -0.4729 & 0.6196 & 0.0131\\ -0.0051 & 0.7924 & 0.6100 & -0.0432\\ 0 & 0 & 0 & 1 \end{array}\right].$$

Secondly, a total of 100 measurements were acquired for the remaining calibration. The navigation robot and operation robot moved randomly within the workspace of the robot under the premise that the OTS can always track the passive tool. In order to verify the effectiveness of the proposed calibration method for dual-robot cooperation systems, the calibration results of the proposed method were compared with Wu’s method [19].

**Figure 4:**
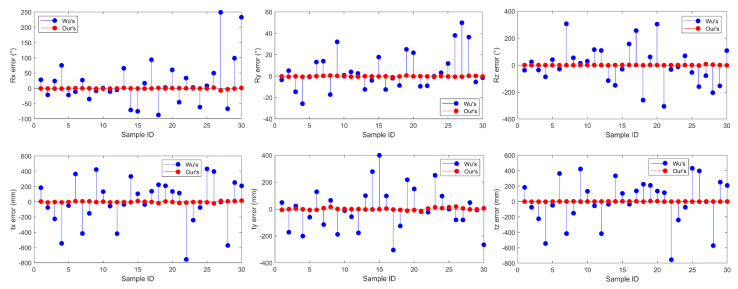
The calibration errors using 30 training data.

In order to compare the sensitivity to the size of the training data, only 30 measurements were used as the training data, and the calibration errors of both methods are provided in Figure 4. For this small amount of training data, Wu’s method cannot converge. Large and severe oscillations can be found on the calibration errors of Wu’s method. On the contrary, the proposed method converges successfully. This verifies the effectiveness and robustness of the proposed method using small amount of data.

**Figure 5:**
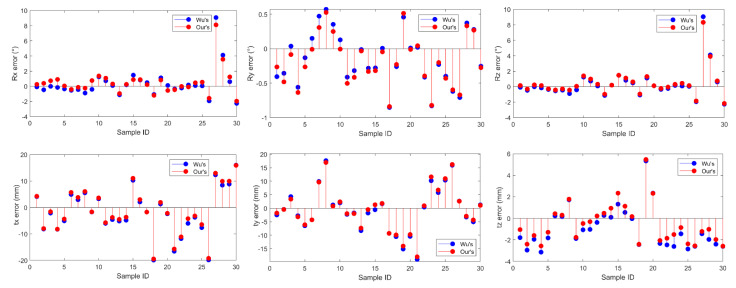
The calibration errors using 100 training data.

If more data are included in the training data, Wu’s method can converge normally, and the calibration error can be made small. For instance, if all the 100 measurements are used as the training data, the calibration accuracy of Wu’s method becomes comparable with the proposed method. Figure 5 shows the corresponding translation and rotation errors. Compared with the calibration results in Figure 4, it can be observed that the calibration errors of both methods were significantly reduced. There exist several samples whose calibration errors are relatively large. This might come from the unexcepted shaking of the robots when the data are acquired in the experiment. The calibrated HTMs of the dual-robot system using the proposed method were:

(33) $$X=\left[\begin{array}{cccc} -0.0224 & 0.6975 & -0.7162 & 0.0444\\ 0.0053 & 0.7165 & 0.6976 & -0.0838\\ 0.9997 & 0.0118 & -0.0197 & -0.0391\\ 0 & 0 & 0 & 1 \end{array}\right].$$

(34) $$Y=\left[\begin{array}{cccc} -0.9999 & -0.0043 & -0.0131 & 2.0005\\ 0.0042 & -1.0000 & 0.0070 & -0.3546\\ -0.0132 & 0.0069 & 0.9999 & 0.0361\\ 0 & 0 & 0 & 1 \end{array}\right].$$

(35) $$Z=\left[\begin{array}{cccc} -0.7798 & -0.3858 & 0.4931 & -0.0603\\ 0.6261 & -0.4729 & 0.6200 & 0.0134\\ -0.0060 & 0.7921 & 0.6103 & -0.0409\\ 0 & 0 & 0 & 1 \end{array}\right].$$

The above experimental results also demonstrate the effectiveness of the proposed method in the calibration of dual-robot systems. It must be noted that few training data are required for the proposed method. This is important in real implementations so as to improve the time efficiency of the calibration.

## 4. Conclusions

The accuracy of the calibration is crucial for the multi-robot collaboration system. This paper proposes an effective method to calibrate the HTMs of hand-eye, flange-tool and robot-robot in a dual-robot collaboration system. The proposed method combines the closed-form method and the iterative method, where the result of the closed-form method is used as the initial value of the iterative method. The simulation results prove that the amount of training data will not affect the calibration effect, and the calibration with high precision can be completed with fewer data. The performance of the proposed method is further verified in the calibration of the prototype of a dual-robot collaboration system developed for orthopedic surgeries. The experimental results show that the translation and rotation errors can be made small using 30 training data, which proves the effectiveness and applicability of the method.

## Figures and Tables

**Table 1:** The basic operations of dual quaternion.

Conjugation	Addition	Multiplication	Magnitude	Inversion
$\begin{array}{c} \;{\hat{q}}^{*}=q^{*}+\varepsilon {q^{\prime }}^{*}\\ \;q^{*}={\left(q_{0},\;-q_{1},\;-q_{2},\;-q_{3}\right)}^{T}\\ {q^{\prime }}^{*}={\left(q_{00},\;-q_{01},\;-q_{02},\;-q_{03}\right)}^{T} \end{array}$	$\begin{array}{l} \;{\hat{q}}_{a}+{\hat{q}}_{b}\\ =(q_{a}+q_{b})+\varepsilon (q{’}_{a}+q{’}_{b}) \end{array}$	$\begin{array}{c} \;\lambda \hat{q}=\lambda q+\lambda \varepsilon q^{\prime }\\ {\hat{q}}_{a}\cdot {\hat{q}}_{b}=(q_{a}\cdot q_{b})+\varepsilon ({q^{\prime }}_{a}\cdot q_{b}+q_{a}\cdot {q^{\prime }}_{b}) \end{array}$	$\left\|\hat{q}\right\|=\sqrt{\hat{q}\cdot {\hat{q}}^{*}}$	${\hat{q}}^{-1}=\frac{{\hat{q}}^{*}}{\left\|\hat{q}\right\|}$

**Table 2:** The range of noise added into the data.

	R_*A*_ (°)	R_*B*_ (°)	R_*C*_ (°)	t_*A*_ (mm)	t_*B*_ (mm)	t_*C*_ (mm)
Noise range	±0.25	±0.5	±0.25	±0.25	±0.5	±0.25
